# DNACPR decisions during Covid-19: An empirical and analytical study

**DOI:** 10.1093/medlaw/fwab047

**Published:** 2022-01-14

**Authors:** Hannah Bows, Jonathan Herring

**Affiliations:** 1 Durham Law School, Durham University, Durham, UK; 2 Faculty of Law, University of Oxford, Oxford, UK

**Keywords:** advance planning, Covid-19, CPR decisions, DNACPR, end-of-life care, resuscitation

## Abstract

Considerable concern has arisen during the Covid pandemic over the use of Do Not Attempt Cardiopulmonary Resuscitation decisions (DNACPRs) in England and Wales, particularly around the potential blanket application of them on older adults and those with learning disabilities. In this article, we set out the legal background to DNACPRs in England and the concerns raised during Covid. We also report on an empirical study that examined the use of DNACPRs across 23 Trusts in England, which found overall increases in the number of patients with a DNACPR decision during the two main Covid ‘waves’ (23 March 2020–31 January 2021) compared with the previous year. We found that these increases were largest among those in mid-life age groups, despite older patients (in particular, older women) having a higher number of DNACPR decisions overall. However, further analysis revealed that DNACPR decisions remained fairly consistent with regard to patient sex and age, with small reductions seen in the oldest age groups. We found that a disproportionate number of Black Caribbean patients had a DNACPR decision. Overall, approximately one in five patients was not consulted about the DNACPR decision, but during the first Covid wave more patients were consulted than pre-Covid.

## I. INTRODUCTION

‘Do not resuscitate’ orders^1^ have achieved a somewhat iconic status in television hospital dramas and in ethical discussions. It is, however, a somewhat misleading term. They are not really orders, for a start, and they do not apply to all resuscitation. They are more properly known as Do Not Attempt Cardiopulmonary Resuscitation (hereafter DNACPR) decisions. The National Health Service (NHS) explains:

CPR stands for cardiopulmonary resuscitation. It is a treatment that can be given when you stop breathing (respiratory arrest) or your heart stops beating (cardiac arrest). CPR tries to get your breathing and heart going again … [Having a DNACPR notice] means if your heart or breathing stops your healthcare team will not try to restart it.^2^

Despite the impression given in the popular media, cardiopulmonary resuscitation (CPR) has a relatively low success rate: around 10%–20% of patients whose heart or breathing have stopped have it restarted following resuscitation.^3^ Even if there is immediate success, ‘only a few people make a full recovery even if their heart or breathing can be restarted with CPR’.^4^ However, as Coleman and colleagues recently pointed out, ‘distinguishing between sudden cardiac arrest, where CPR is more likely to be successful, and cardiac arrest in the context of a deterioration is difficult’, and, therefore, ‘standard practice is usually to attempt CPR on any inpatient who has a cardiac arrest, regardless of the underlying cause’.^5^

CPR carries risks of bruising, broken ribs, and punctured lungs. It can be distressing to experience and witness and, in many cases, is ‘at best futile and at worst degrading and physically harmful to a patient in the dying phase’.^6^ This means that there must be a careful weighing up of the possibility of the benefits of CPR (the treatment) against the potential and, in many cases inevitable, harms.

The decision not to resuscitate a patient can arise in one of two circumstances: either as part of advance planning or where the need for resuscitation arises at a crisis moment. In the latter situation, there may well not be time to fully assess the patient’s background, history, and underlying health before starting resuscitation. In the former, and where it is foreseen that a patient may suffer respiratory or cardiac arrest in the future, it may be preferable for the team to make a carefully considered decision in advance, taking into account the health, wishes, and well-being of the patient.^7^ This avoids the resuscitation decision being made rapidly, in an emergency situation. Thus, it is important to have discussions with patients with long-term conditions about advance care, emergency treatment, and end-of-life decisions, in order to support ‘upstream’ planning, including whether a patient would like to receive CPR should they be in a situation that potentially warrants this intervention.^8^ DNACPRs are an important mechanism for discussing and recording patient decisions in relation to CPR, as part of a ‘broader spectrum of care planning for long term conditions, advance care planning for end-of-life decisions, and emergency treatment escalation plans’.^9^

The Master of the Rolls in *R (Tracey) v Cambridge University Hospitals*, highlighted the practical significance of DNACPRs:

DNACPR orders are likely to affect most of the population directly or indirectly. According to evidence that we have been shown, 68% of the population die in hospital and 80% of these die with DNACPR notices in place. In other words, in relation to more than 50% of the population, a decision is taken in advance of their deaths that, if they are subject to a cardio-pulmonary arrest, they will not receive cardiopulmonary resuscitation (‘CPR’).^10^

Considerable concern has arisen during the Covid pandemic over the use of DNACPRs in England and Wales.^11^ In particular, the media and some care providers claimed that older people and those with intellectual differences had DNACPRs placed on their notes without consultation with them and/or their family, and that these decisions were made about patients simply due to age or intellectual ability.^12^ These concerns may have been magnified due to the restrictions on families and friends visiting people in care residence settings. This may have hindered the possibility of involving families in decision-making and meant that families were unclear what decisions had been made concerning their loved ones. In the remainder of this article, we outline the concerns that arose about the use of DNACPRs during Covid and the potential legal ramifications of these (Section II). Having set out our methods in Section III, we report on an empirical study which examined the use of DNACPRs across 23 Trusts in England (Section IV), which found overall increases in the number of DNACPR decisions during the two main Covid ‘waves’ (23 March 2020–31 January 2021) compared with the previous year.

## II. COVID AND DNACPR DECISIONS

Two broad concerns about DNACPR decisions were raised during the height of the Covid-19 pandemic. First, that such decisions were made without proper consultation with patients and/or their families.^13^ At the same time, Compassion in Dying reported receiving a significant increase in calls during the pandemic, with most of these from people wanting to ‘protect themselves’ from having CPR against their wishes.^14^ It seems, therefore, that there were issues both with the creation and non-creation of DNACPR decisions.

There are two separate reasons why a failure to consult with the patient and/or their relatives or carers before placing a DNACPR may be unlawful. First, the case law is clear that there is a duty to consult with a patient before putting a DNACPR on their medical record. In *Tracey*,^15^ the Court of Appeal held that a failure to consult with a patient before placing a DNACPR decision on their record amounted to a breach of the patient’s right to respect for their private life under Article 8 of the European Convention on Human Rights (ECHR).^16^ Lord Dyson stated that:

A DNACPR decision is one which will potentially deprive the patient of life-saving treatment, there should be a presumption in favour of patient involvement. There need to be convincing reasons not to involve the patient.^17^

The only example of a convincing reason, he suggested, was if doing so would cause the patient psychological harm, but he added that **‘**doctors should be wary of being too ready to exclude patients from the process on the grounds that their involvement is likely to distress them’.^18^ He noted that discussing the end of life was likely to bring distress, and so more than that kind of distress was required to invoke this reason. Even where this ‘exception’ applies, that does not seem to be a reason not to discuss the issue with the patient’s relatives. Indeed, where a patient is unable to participate in a consultation, they have a right under Article 8 for their family members to be consulted, where it is practical and appropriate to do so.^19^ It is worth noting that the failure to consult with family members or carers breaches the *patient’s* Article 8 rights, because the purpose of consulting with relatives is to ensure that an appropriate best interests assessment of the particular patient can be made, rather than to protect any interests of the relatives themselves.

Importantly, in *Winspear v City Hospitals Sunderland NHS Foundation Trust* Blake J stated that:

The fact that there was no cardiac arrest before the notice was cancelled is not decisive, as its existence is itself an interference with private life; it is an important decision about medical treatment of a potentially life-saving nature.^20^

This makes it clear that it is the *issuing* of the DNACPR, rather than not supplying treatment, which causes the breach of Article 8. This is important because in many cases it would be difficult to prove that without the DNACPR the resuscitation would have been provided, making any legal claim hard to establish. *Tracey* and *Winspear* have had a significant impact on the way DNACPR were made in many hospitals.^21^

The second reason why a failure to consult may be unlawful is that, as already indicated, it will not be possible to make a proper best interests assessment regarding a patient without consultation with them and/or their family and carers.^22^ In such a situation, doctors will have to ‘guess’ at what values and principles play into the best interests assessment of a particular patient. And blanket policies or assumptions based on old age and intellectual ability will mean that an individual assessment has not been made. These dangers are acknowledged by the NHS:

DNACPR decisions should not be made for a group of people at once. For example, DNACPR decisions should not be made for everyone living in a care home or for a group of people over a certain age. This is unlawful, irrespective of medical condition, age, disability, race or language. Learning disability, autism or dementia are not reasons to put a DNACPR on someone's record.^23^

The second concern raised by the media and individuals during Covid was that DNACPR decisions were being applied to groups of people, rather than there being a proper consideration of *individual* circumstances. They were, thus, discriminatory,^24^ and the use of age or intellectual abilities to make blanket policies on DNACPRs, or as the sole factor in making a DNACPR for a particular patient, would be readily open to legal challenge. For a start, it would be contrary to the NHS’ own guidance that a decision must be made on an individual basis.^25^ A declaration could thus be sought that the application of the DNACPR was unlawful, most probably under the inherent jurisdiction or the Mental Capacity Act 2005 if the patient lacked the capacity to make decisions for themselves. An application for judicial review could also be brought if the blanket policy was produced by a trust.^26^ Such a policy would amount to discrimination on the basis of age and disability, and could be challenged under the Equality Act 2010 or the Human Rights Act 1998.^27^ In all these cases, the primary remedy would be a declaration that the DNACPR was unlawful. If the DNACPR was acted upon and a patient was not given CPR, then an action could lie in negligence or even, potentially, for gross negligence manslaughter.

The concerns about the blanket application of DNACPRs on groups of people and imposing DNACPRs on patients without consultation, were sufficient to lead the Department for Health and Social Care (DHSC) to commission the Care Quality Commission (CQC) to undertake a review of practice during Covid, which took place between November 2020 and January 2021.^28^ The review looked at a wide range of health settings, from care homes to hospitals. The CQC consulted with 50 external stakeholders, surveyed 2,048 adult social care providers, 613 patients with a DNACPR and/or their families, reviewed 166 DNACPR records, and held 156 interviews or focus groups with clinicians. Two key issues emerged from the review. First, the CQC found significant variation in the involvement of people in decisions about their care; in particular, over DNACPRs. While some people reported that they felt that they had been involved in the decision-making process, others felt those conversations had come ‘out of the blue’ and that they were not given the time or information to fully understand what a DNACPR was. The report highlighted that older people and those with learning difficulties were particularly unsupported. In some cases, people were not aware that a DNACPR decision was in place, which the CQC noted ‘could be hugely distressing for people and their families and/or carers’.^29^

Secondly, the CQC also examined whether there was evidence that DNACPR decisions were being made about *groups* of people, with ‘blanket’ DNACPR decisions applied without consideration for individual patients. The CQC found that although ‘most providers’ were unaware of such blanket policies, there was evidence from families and carers that they were used in some cases, which ‘risks undermining public trust and confidence in the health and care system and demonstrates the need for better oversight of DNACPR decisions’.^30^ The CQC concluded that the inability to find the extent of blanket DNACPR was because of poor record keeping. It was thus not possible to determine the extent to which personalised decisions had been made, rather than discriminatory assumptions being used. They noted the wide range of advance care planning forms in use, differences in procedure, and a lack of consistency and confusion in the language used during conversations with patients.

The CQC report is the largest report into DNACPRs during Covid, but a number of other smaller-scale reports have been produced.^31^ The Queen’s Nursing Institute Survey of Care Homes^32^ found that 16 out of 163 care homes reported negative changes to the use of DNACPRs as a result of Covid, including ‘instructions from the GP or the CCG or hospitals putting DNACPR in place without discussion with the resident, family or care home’.^33^ The British Institute of Human Rights also undertook an investigation into the use of DNACPRs in 2020, through a survey of 230 individuals with support needs and/or their families, friends and carers, advocates and community groups, and staff working in care and support services.^34^ The report sets out some concerning examples, including that:

According to a care provider, three services (in Somerset, Derbyshire and East Sussex) were contacted by GPs to say that they have deemed the people they support, who have learning disabilities, and other complex needs, should all be DNR. There was no mention of consultation with families or best interests’ assessments.^35^

A report by Compassion in Dying likewise listed some of the reports that they had received; for example, ‘A hospital doctor told me it was routine hospital procedure for anyone over the age of 70 to have a DNACPR placed on them’.^36^

While these reports are anecdotal and hard to verify, the number of incidents listed in these reports is sufficient to raise serious concerns.^37^ At the same time, it is important to remember that Compassion in Dying stated that the majority of calls to them during the pandemic were from individuals seeking to protect themselves from CPR.^38^ Moreover, a recent study examining DNACPRs in patients admitted to hospital with suspected Covid-19 during the first wave, reported that although almost a third of patients (3,929/12,748) had a DNACPR (which is higher than the rates reported in previous studies of conditions similar to Covid-19),^39^ this did not impact on life-saving treatment.^40^ In other words, people with a DNACPR received intensive treatments as frequently as those without one.

These studies and regulator inquiries shed light on a number of issues with the use and application of DNACPRs during Covid-19 in the UK and/or wider (mis)understandings about the appropriateness of them. Yet, there remain gaps in the evidence concerning the *difference*s in DNACPR practice during Covid compared with pre-Covid times, as well as the characteristics of patients who have a DNACPR decision. In the remainder of this article, we present our findings from a rapid snapshot study that examined the use of DNACPRs before and during the two main Covid-19 waves, with a focus on the demographic characteristics of patients with a decision in place. This allowed us to examine whether there was any difference in the overall number of DNACPRs, and the profile of patients with such a decision during Covid.

## III. METHODS

In this study, we examined the use of DNACPRs between 23 March 2019 and 31 January 2021, and compared the overall number and demographics of patients with a DNACPR during the two main pandemic waves (23 March 2020–1 August 2020 and 1 October 2020–31 January 2021) with the same periods in the previous year. Freedom of Information (FOI) requests were sent to all Foundation and Hospital Trusts in England (*n* = 313). The Freedom of Information Act 2000 provides the legal basis for individuals to access data held by public bodies, placing ‘significant duties and responsibilities on public authorities to give access to the information they hold’.^41^ Public bodies include a range of organisations and institutions, such as government departments, police, universities, councils, and health trusts. Under the 2000 Act, public bodies are required to release the data requested (if it is held by the organisation or agency) within 20 working days, unless one or more of the exemptions are engaged. The FOI Act can, thus, provide ‘access to a significant amount of information for a variety of research purposes’,^42^ particularly for research into healthcare organisations such as NHS Trusts, which hold vast amounts of data that can be accessed with relative ease and speed when compared with other methods that often require lengthy negotiations.

However, while FOI requests provide rapid access to publicly held data, the method is limited in a number of ways.^43^ Of particular relevance to this study is the requirement that the data requested do not relate to personal information (section 40(2)) and/or do not breach confidentiality (section 41). This means that FOI requests must be designed in a way that is compliant with the UK General Data Protection Regulation and the Data Protection Act 2018. Thus, data on living individuals cannot usually be accessed using FOI requests, and such requests can usually only be used to gain access to aggregated data.

Our FOI request comprised five questions. Questions 1–4 asked for the total number of DNACPR decisions made during four periods: 23 March 2019–1 August 2019 (Period 1); 1 October 2019–31 January 2020 (Period 2); 23 March 2020–1 August 2020 (Period 3); and 1 October 2020–31 January 2021 (Period 4). Question 5 asked for aggregated demographic data for each period as follows: patient sex/gender; patient age category (16 or under; 17–24; 25–29; 30–34; 35–39; 40–44; 45–49; 50–54; 55–59; 60–64; 65–69; 70–74; 75–79; 80–84; 85–89; 90 or above); patient ethnicity (as per government groups);^44^ diagnosed learning disability (number of patients with a diagnosed learning disability); and number of DNACPR decisions issued with and without patient consultation or knowledge. Finally, we asked for the number of patients who died within eight weeks of a DNACPR, so we could examine the proportion of patients who died within a relatively short time following a DNACPR decision being issued. Ethical approval for the study was not required because FOI requests provide access to data considered prima facie public and so is not typically subject to ethical approval.^45^

In total, 26 Trusts (8%) responded fully or partially to the request. The majority of Trusts declined the request, engaging the section 12 time/cost exemption on the basis that the information requested on DNACPRs was not centrally held and that to retrieve it would require manual examination and extraction from individual patient records, exceeding the time/cost threshold set out in the FOI Act (18 hours/£450 for non-government public bodies). For example, one Trust responded that ‘in order to provide this information, a manual check of paper clinical records would be required equating to 52,500 sets of notes’. The Trusts who complied with the request had moved to centralised electronic patient record systems, which allowed the material requested to be searched for and extracted within the time/cost limit. This underscores the importance of having digital patient and health data systems,^46^ which NHS England has declared to be a key objective but has yet to be realised.^47^

Three Trusts provided data in a way that could not be analysed, primarily because they gave an indication or range rather than a specific figure. For example, instead of specifying how many patients were aged under 16, they indicated that there were less than five (<5). The remaining Trusts (*n* = 23) included in this study provided some or all of the data in a format that could be combined and analysed. The Trusts ranged in both size and geographical location, meaning that some Trusts provided data relating to thousands of DNACPRs, whereas others had only a small number (<50) of DNACPRs recorded across the four periods. As such, comparisons between the different Trusts cannot be made. Instead, the data were combined and inputted into Microsoft Excel where descriptive (for example, overall counts/frequencies, mean, median) and comparative analyses were performed.^48^

This study has a number of limitations. The method, FOI requests, provides a useful tool for researchers to gain rapid access to public data not otherwise available. However, FOIs are not primarily designed for research purposes and the amount and type of data received is restricted by a number of exemptions contained within the FOI Acts. Consequently, although large amounts of data were accessed in this study, the data were provided in aggregated form. This prevents individual-level patient analysis of DNACPR decisions and means that further detailed examination of the relationship between different patient characteristics is not possible. Moreover, no narrative data can be captured through this method, so it was not possible, for example, to gain insight into the reasons why some patients were not consulted about a DNACPR decision. Nevertheless, this study provides insights into DNACPR practice before and during Covid-19 for a large number of patients in England, which can be used to inform further research and discussion in this area.

A further limitation is the small number of Trusts who were able to comply with the data request, meaning that data from individual Trusts could not be compared with other Trusts and, instead, had to be combined. The findings, thus, cannot be generalised.

## IV. FINDINGS

Across the 23 trusts who provided usable data, there were 126,545 DNACPR decisions issued between 23 March 2019 and 31 January 2021 (Table 1). When comparing the overall number of decisions issued during the two peak Covid waves in England with the same periods in the previous year, there was an increase of 30% for the first wave (Period 3) compared with the same period in 2019 (Period 1), and an 11% increase for the second wave (Period 4) compared with the same period in 2019–2020 (Period 2). Thus, the increase during the second wave was more modest than that in the first.

**Table 1:** : Overall number of patients with a DNACPR decision across twenty-three Trusts broken down by data period

Data period	*N*
Period 1 (23 March 2019–1 August 2019)	27,712
Period 2 (1 October 2019–31 January 2020)	29,801
Period 3 (23 March 2020–1 August 2020)	35,952
Period 4 (1 October 2020–31 January 2021)	33,080
Total	126,545

In total, 22 Trusts provided a breakdown of patient sex across the four periods (*n* = 126,163). Overall, more women than men had a DNACPR (Table 2); 68,520 (54%) of patients with a DNACPR were female, compared with 57,643 male patients (45%). In a small number of DNACPRs (*n* = 13 across two Trusts), patient sex had not been recorded. The sex breakdown of patients remained broadly consistent across the four data periods, with a slight decrease in the proportion of female patients during Covid. During Period 1 and Period 2 (pre-Covid), female patients accounted for 55% of DNACPRs, whereas during the first Covid wave (Period 3) females accounted for 53% and during the second Covid wave (Period 4) they accounted for 54%.

**Table 2:** : Overall number of patients with a DNACPR across twenty-two Trusts broken down by sex and data period

Data period	*N*	Male	Female	Unknown
Period 1 (23 March 2019				
–1 August 2019)	27,662	12,345 (45%)	15,317 (55%)	1 (<1%)
Period 2 (1 October 2019				
–31 January 2020)	29,751	13,270 (45%)	16,481 (55%)	7 (<1%)
Period 3 (23 March 2020				
–1 August 2020)	35,799	16,761 (47%)	19,038 (53%)	0
Period 4 (1 October 2020				
–31 January 2021)	32,951	15,267 (46%)	17,684 (54%)	5 (<1%)
Total	126,163	57,643 (45%)	68,520 (54%)	13 (<1%)

**Figure 1:**
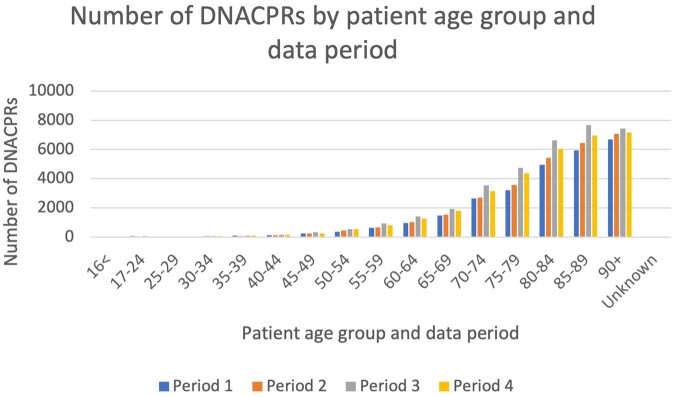
Number of DNACPRs by patient age group and data period

**Figure 2:**
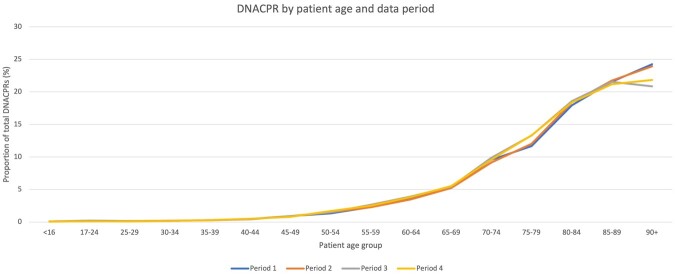
Proportion of patients with a DNACPR by age group and data period

With regards to age, 20 Trusts provided age data on patients issued with a DNACPR across the four periods (*n* = 125,530), although in seven DNACPRs the age of the patient was unknown/not recorded. The majority of patients were aged over 80 (*n* = 78,420, 62%), and, in general, the number of DNACPRs increased with patient age, with relatively few (*n* = 1,119, 0.9%) involving patients aged under 40. Trusts were asked to provide the age breakdown for each data period, which was analysed to examine whether there were differences in the age groups of patients with a DNACPR during Covid compared with the previous year (Fig. 1). During the first Covid wave (Period 3), increases in the number of DNACPRs were seen across all age groups, with the largest increases observed in the age groups 30 to 34 (55% increase on Period 1), 55 to 59 (50% increase on Period 1), 75 to 79 (47% on Period 1), and 50 to 54 (47% increase on Period 1). However, when looking at the overall proportion of DNACPRs by age group during the first wave (Period 3) compared with the previous year (Period 1), the picture remains broadly similar (Fig. 2). In other words, the age profile of patients with a DNACPR did not differ during the first Covid wave (Period 3) compared with the previous year (Period 1).

During the second wave (Period 4), there were decreases observed in the number of DNACPRs in some age groups compared with the previous year (Period 2). For example, for patients aged 16 or younger there were 17 DNACPRs during the second Covid wave (Period 4) compared with 32 before Covid (Period 2). Decreases were also observed in age groups 25 to 29 and 45 to 49, and there was no change for the age group 17 to 29. Again, the overall proportion of DNACPRs by age group remained broadly similar (Fig. 2). However, those in the oldest groups (85 to 89, and 90+) made up proportionally fewer of the DNACPRs during the two Covid waves (Periods 3 and 4) compared with the previous years (Periods 1 and 2).

**Figure 3:**
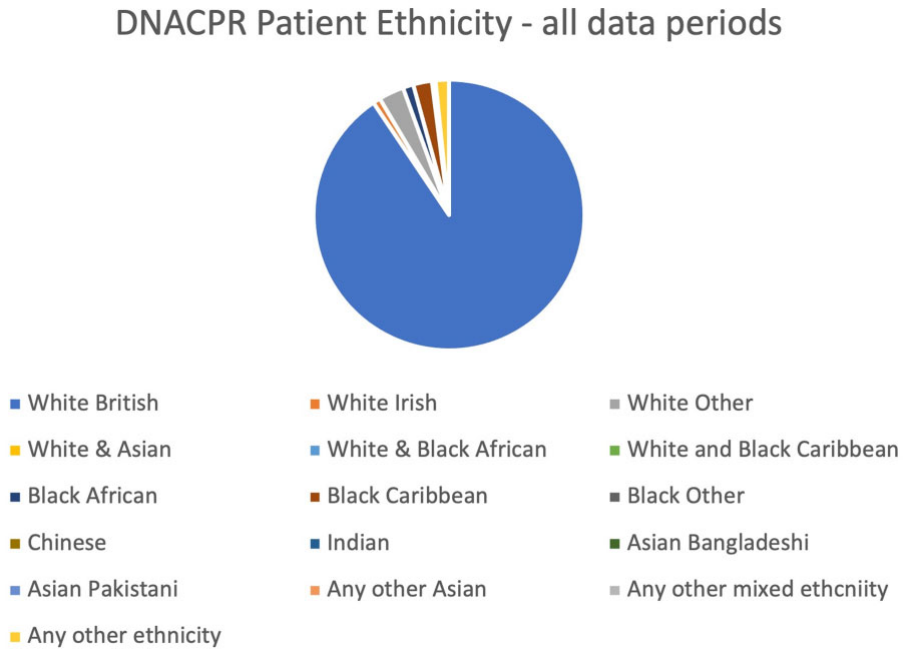
Proportion of patients with DNACPR across all periods by ethnic group

Trusts were also asked to provide a breakdown of patient ethnicity. Eighteen Trusts provided data that could be analysed, corresponding to 95,531 patients (Fig. 3). In 10,677 cases, there was no record of patient ethnicity or it was ‘not stated’ or ‘unknown’, leaving 84,854 where patient ethnicity could be assessed. White patients accounted for 92% of patients overall, made up of ‘White British’ (88%), ‘White Irish’ (0.87%), and ‘White Other’ (3%). Black patients accounted for just under 4% of patients. Black Caribbean patients accounted for 2% of patients with a DNACPR, Black African for 1%, and ‘Black Other’ for 0.41%. No meaningful differences in relation to the ethnicity of patients with a DNACPR were observed during the two Covid wave data periods (Periods 3 and 4) compared with the pre-Covid data periods (Periods 1 and 2).

**Figure 4:**
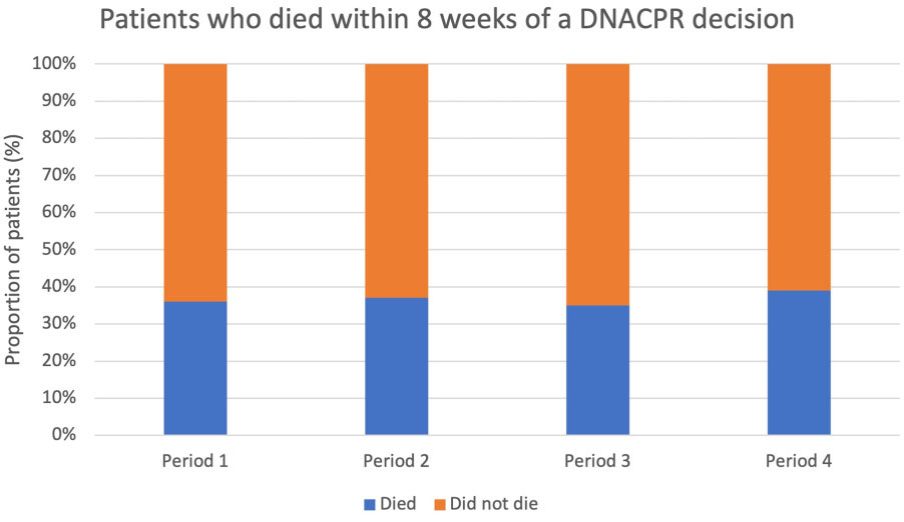
Overall number of patients who died within 8 weeks of a DNACPR decision

Eighteen Trusts provided data on how many patients died within eight weeks of a DNACPR being issued, relating to 89,103 patients (Fig. 4). Overall, the proportion of patients who died within eight weeks of a DNACPR remained broadly similar across the four periods examined, ranging from 36% in Period 1 to 39% in Period 4. When comparing the lockdown periods with the same time in the previous year, there was a small decrease in the number of patients who died within eight weeks of a DNACPR in Period 3 (35%) compared with the same time in Period 1 (36%), and a small increase during Period 4 (39%) when compared with Period 2 (37%).

As to the number of patients issued with a DNACPR who had a learning disability, 14 Trusts provided data in an accessible format, concerning 68,884 patients with a DNACPR between 23 March 2019 and 31 January 2021. In total, 622 (0.9%) had a registered or diagnosed learning disability. There was no change in the proportion of patients with a DNACPR who had a learning disability during the two pandemic waves (Periods 3 and 4) compared with the pre-Covid data periods (Periods 1 and 2).

**Figure 5:**
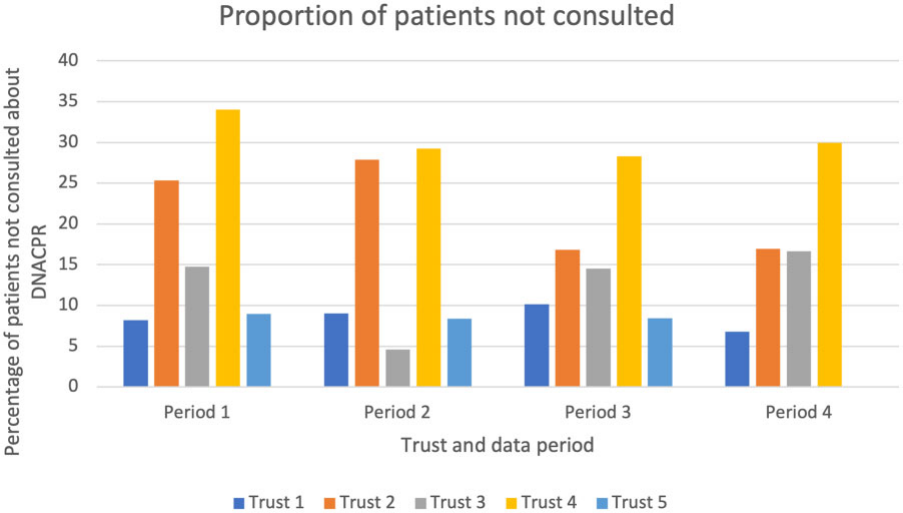
Proportion of patients not consulted about DNACPR decision by Trust and data period

Finally, Trusts were asked to provide data on the number of patients who were issued a DNACPR without consultation. Only five Trusts provided this data, relating to 14,167 patients. On average, one in five (*n* = 2,835, 20%) patients were issued with a DNACPR without consultation. Data on whether other individuals (such as family members) were consulted were not provided. During the first Covid wave, the proportion of patients with a DNACPR who had not been consulted was lower than in the previous year; 16% of patients during Period 3 compared with 18% of patients during Period 1. Variation in the proportion of patients who had not been consulted about the DNACPR decision was observed across the four data periods and between Trusts (Fig. 5). For example, pre-Covid (Period 1 and Period 2) Trust 2 did not consult patients in 25% and 28% of cases, respectively, but this fell to 17% during both Covid waves (Periods 3 and 4). Conversely, pre-Covid (Period 1 and Period 2) Trust 1 did not consult patients in 8.18% and 9% of cases, respectively, and this increased to 10.15% during the first Covid wave (Period 3) but then fell to 6.75% during the second Covid wave (Period 4) (Fig. 5).

## V. DISCUSSION

Our study examined data from 23 English NHS Trusts of varying size and location relating to the number of DNACPR decisions made before and during the two main Covid-19 waves in England. We found an overall increase in the number of DNACPR decisions during both Covid-19 waves (Periods 3 and 4) compared with the same periods a year earlier pre-Covid (Periods 1 and 2). Our research thus confirmed media and anecdotal reports of increases in DNACPRs during the pandemic. Our findings are consistent with the only other published study on DNACPR practice during Covid-19, which examined decisions recorded at a single teaching hospital.^49^ However, that study found that the rate of new DNACPR forms during Covid-19 (1 March to 30 April 2020) doubled compared with the analogous period in the previous year, whereas we observed smaller increases of 30% during the first wave (Period 3) and 11% during the second wave (Period 4).

In our study, we found that there were more women with DNACPR decisions on their medical records than men, both before and during Covid-19. However, as most patients across all four data periods examined were older (a majority were aged over 80), this finding is not unexpected as women have a higher life expectancy than men and account for 55% of adults aged 80,^50^ and over two-thirds of the population aged 90 and above.^51^ We found that, overall, 55% of patients with a DNACPR before Covid-19 were female, and that this reduced slightly to 53% during the first wave and 54% during the second wave. Our findings are consistent with those of Coleman and colleagues, who found that females accounted for 52.1% of DNACPRs pre-Covid but that this reduced to 46.9% during Covid-19.^52^

One of our interesting findings was that the biggest percentage increases in DNACPRs during the two Covid-19 waves were observed among mid-life age groups—in contrast to much of the media reporting which focused on the (alleged) disproportionate use of DNACPRs involving older people during the pandemic. During the first wave (Period 3), those aged between 30 and 34, and 55 and 59 saw the biggest increases, while those in the oldest (90+) and second youngest (17 to 24) groups saw the smallest. During the second wave (Period 4), those aged 40 to 44 and 50 to 54 saw the biggest increase, while among patients aged 16 and under and 25 to 29 the number of DNACPR decisions decreased compared with Period 2. However, when looking at the overall proportion of patients with a DNACPR by age group during the two Covid waves (Periods 3 and 4) compared with the previous periods (Periods 1 and 2), we did not observe meaningful differences, with the exception of the oldest patients (aged 90 and over). In other words, the age (and sex) profile of patients with a DNACPR remained broadly the same during Covid as pre-Covid.

Again, Coleman and colleagues reported similar findings, with the proportion of patients aged under 66 with a DNACPR increasing from 21.9% during March–April 2019 to 32.3% during March–April 2020.^53^ Thus, widespread concerns about the potential illegal practice of blanket DNACPRs based on patient (old) age (see Section II) *are not* supported by our findings. However, a simple quantitative assessment of the number of DNACPRs issued by age group over a particular period does not give any indication of the quality, or legality, of those decisions. It is therefore critical that future research examines the decision-making underpinning DNACPRs, during Covid and more generally, to ensure that decisions are being made in line with both guidance^54^ and with legal obligations under the Equality Act 2010 and Human Rights Act 1998.

Over the four data periods we examined, we found that although the majority of patients with a DNACPR decision were white, a disproportionate number of Black Caribbean patients had a DNACPR. Overall, 2.19% of DNACPRs involved a Black Caribbean patient, whereas this ethnic group accounts for only 1.1% of the national population.^55^ This discrepancy requires further analysis to explore whether it is observed nationally, as our sample was not representative. This finding may thus reflect the population demographics of the Trusts which responded to the data request.

Having said that, interestingly, the CQC investigation found that people from a Black or minority ethnic background were most likely to feel involved in conversations about medical treatment and DNACPRs.^56^ Meanwhile, a recent analysis of DNACPRs among Covid-19 patients reported that people of an Asian ethnicity were less likely than those from other ethnic groups to have an early (before or on the day of hospital admission) DNACPR decision.^57^ Combined, these studies and our findings indicate that ethnicity may be an important factor in DNACPR policy, and practice, and so should be a focus of local and national audits and research on DNACPRs to establish whether patients from ethnic minority groups are disproportionately represented in DNACPR decisions. If they are, it is important to understand what is driving this over-representation. If the use of DNACPRs does reflect unequal treatments on the grounds of ethnicity, this might raise issues under the Equality Act 2010, particularly whether there is a breach of the public sector equality duty under section 149. There may also be claims under section 2 of the Health and Social Care Act 2012, which requires NHS Trusts to have regard to the NHS Constitution where the right not to be discriminated against on the grounds of race is protected.^58^ This could be enforced through a claim in the tort of negligence or an application for a judicial review.

Contrary to media reporting of DNACPRs during the pandemic, we found that a very small proportion of patients with a DNACPR had a diagnosed learning disability. Indeed, less than 1% of the patients reported from the trusts had a learning disability across all four data periods. We are not denying that there have been cases where those with learning difficulties have discriminated against in relation to DNACPRs, but it does not appear to be an NHS-wide problem. Thus, concerns that blanket DNACPR policies were illegally applied based on intellectual ability/a learning disability (see Section II) were not borne out in our analysis. This finding appears to fit with those of the CQC, who found that for people with a learning disability and autistic people, 76% (16 out of 21) and 94% (17 out of 18) of individuals, respectively, felt that their best interests and capacity were completely or mostly considered. Furthermore, 70% (69 out of 98) and 81% (57 out of 70) of relatives or carers answering on behalf of individuals, said that the person’s best interests and capacity had been completely or mostly taken into consideration.^59^ This compares with their more general public survey, which found that only 70% (48 out of 69) of individuals with a DNACPR decision felt that their best interests and capacity were completely or mostly considered.^60^

Our findings indicate that in approximately 14,167 patient DNACPRs, around one in five patients had not been consulted. However, when looking at practice during and before Covid, we found that the proportion of patients who were not consulted about the DNACPR decision actually fell during the two Covid waves. In other words, *more* patients appear to have been consulted about the DNACPR decision during Covid than in the two periods (Periods 1 and 2) before Covid. The reasons why DNACPRs are not discussed with patients can be complex. In some cases, it is not possible; for example, if the patient is not conscious or lacks capacity. In situations where a patient is deemed to lack capacity, guidance from the General Medical Council states that the professional considering a DNACPR decision should consult with any legal proxy who has authority to make the decision for the patient or, if no legal proxy exists, the issues should be discussed with those close to the patient. This includes family and the healthcare team.^61^ It may have been that other family members or next of kin had been consulted, but these data were not provided in our study. It is of note that our findings are higher than those in the CQC review, which reported that just over 5% of DNACPRs were made without patient or family/next-of-kin consultation,^62^ as well as in academic analysis of practice during the pandemic, which reported that almost 96% of DNACPR discussions involved a patient.^63^

Our findings are, however, broadly consistent with an earlier review of DNACPR decisions at a single Trust in 2016, which found that only 71.4% of patients were involved in the decisions—although 100% of those not discussed with patients were discussed with their family.^64^ Our finding that, overall, a fifth of patients were not directly involved in a DNACPR decision suggests that further examination of DNACPR practice during and post-Covid-19 is required, to ensure that DNACPR decisions are within legal guidelines and patient-centred frameworks.

### A. Legal Implications of the Findings

Failing to consult up to a fifth of patients about a DNACPR decision may indicate an extensive breach of the human rights of patients. As mentioned in Section II, the *Tracey* litigation made it clear that consultation with a patient and/or their family before placing a DNACPR decision on a patient’s medical record is an important aspect of the right to respect for private and family life under Article 8 of the ECHR.^65^ However, it is also worth noting that if the failure to consult has not led to the patient suffering harm, it is unlikely that extensive damages will be awarded by the court.^66^

We suggest that greater attention be paid to the basis of a DNACPR decision. There can be a number of different legal justifications behind a DNACPR, and, in some circumstances, the legal basis can be significant. The following are the primary legal basis for a DNACPR, only one of which is required for an effective decision. First, cases where a court order has been made determining that it would not be in the best interests of a patient who lacks capacity to make the decision to receive resuscitation treatment. In such a case, a DNACPR decision may be placed on their notes to ensure compliance with the order. In *County Durham & Darlington NHS Foundation Trust v PP*, an order was made:

That attempts at resuscitation in the event of either a cardiac or respiratory arrest are likely to cause harm to P, which may have terminal or other deleterious consequences, such that it would not now be in her interests that they be attempted.^67^

In such a case, it would be contempt of court to perform CPR in the circumstances referred to in the order. It should be emphasised that it would be the breach of the court order which would be the legally significant fact in such a case. The existence of a DNACPR decision would simply have the role of alerting the medical team to the existence of the court order.

Secondly, cases where medical professionals have decided in advance that CPR would not be clinically in the best interests of the patient should the need for it arise. This may be because the patient’s underlying health state is such that CPR would have no or minimal chance of success, and/or that due to the patient’s state of health the performance of CPR would cause them serious harm, and/or that their continued life would be of such low quality that the treatment would not be beneficial. The NHS website explains:

your doctor may think that CPR will not help you live longer or that giving you CPR could cause you more harm. This may be because your organs are already too damaged because of another illness or you are approaching the end of your life.^68^

Thirdly, there may be cases where a patient has issued an effective and applicable advance decision that they do not wish to receive resuscitation; for example, *NHS Cumbria CCG v Rushton*.^69^ In such a case, as long as the advance decision *is* effective (for example, it has not been revoked) and *is* applicable (for example, it refers clearly to the treatment in question) then it is binding on the healthcare professionals, even where they think that CPR would benefit the patient. A DNACPR may thus be used on the patient’s notes to ensure that the advance decision is complied with. In such a case, it is the advance decision (rather than the DNACPR decision) that is legally significant.

Finally, cases where it is not in the best interests of a patient, broadly understood and taking into account their wishes and values, to receive CPR. Normally, this will be on the basis that the patient’s quality of life, even if the CPR was successful, will be so low that CPR will not benefit them. This might include a case where although the patient has not made an advance decision, formally defined, they have made it clear that they do not want to continue living or their values are such that it would not be in their best interests to continue living. This assessment may not be based primarily on medical issues, but may focus on the particular wishes and values of the patient.

Of course, in some cases, a combination of these factors will be relevant, but distinguishing these features is important. Where, for example, there is an advance decision to refuse CPR, that may not be provided *even if it is clinically appropriate*. By contrast, if the DNACPR is based on a prediction about what will be clinically appropriate, it may be departed from if the clinical assessment at the time is that CPR *is* appropriate. The basis of the DNACPR is also important if the patient is at the time requesting CPR. That request may be seen as invalidating an advance decision or a best interests decision based on the previous views of the patient. It does not, however, invalidate a DNACPR based on a clinical assessment. These distinctions are significant in discussions with patients and/or relatives.

Having said that, one possible explanation for the concern around a lack of consultation with patients about a DNACPR during the pandemic may be that health professionals asking a patient whether they want to receive CPR in the future was seen as implying a clinical assessment that CPR would be inappropriate. It is thus important that discussions distinguish between whether CPR should be offered to the patient as an appropriate treatment, and whether it is in their best interests (bearing in mind their views and values) to receive CPR. Such discussions will also provide essential information about the beliefs, values, and character of the patient, all of which are essential to a contemporary understanding of best interests under section 4 of the Mental Capacity Act 2005.

The controversy around DNACPRs centres on the final case above, where the team has determined that even though CPR might be clinically justifiable, it is not in the best overall interests of the patient. Cases where the basis of the DNACPR decision is that the patient’s quality of life is so low that it will not be beneficial to preserve the life, can be particularly contentious. The difficulty here is that assessments of a person’s quality of life depend on a wide range of moral, religious, and personal values. That is why in *Aintree University Hospital NHS Foundation Trust v James*, when discussing issues around withdrawing life-sustaining treatment, Lady Hale emphasised the importance of considering the issue from the perspective of the individual patient.^70^ That is only possible if clinical team discusses the issue with the patient and/or their relatives.

### B. Policy Implications of the Findings

One of the limitations of our study (detailed in Section III) is that a relatively small number of Trusts (8% of all Trusts in England) responded with some or all of the data requested. This limitation is a finding in itself, as the majority of Trusts who refused the data request did so on the basis that it was not possible to provide the data within a reasonable time (as set out by the FOI Act) because DNACPR data were not centrally recorded and would require manual examination of individual, paper-based patient records. We therefore echo previous calls to move towards digital patient records and centralised DNACPR recording systems,^71^ and we recommend improving record keeping on DNACPRs in this way to facilitate easy access to information on the use of DNACPRs. The NHS has committed to a digital transformation which includes moving to paperless, integrated electronic health record systems.^72^ Originally, the NHS’ ambition was for this to be achieved by 2020, but our study has revealed that the vast majority of Hospital and Foundation Trusts have not fully realised this ambition. Recent research that audited the implementation of such a system for the storing of DNACPR records has highlighted the potential value of wider adoption of these systems, which the authors conclude is important to ensure that information ‘can then be translated into a wider understanding of DNACPR decisions and advanced care planning for patients both in the community and in hospital’.^73^

We agree that it is critical that robust data on DNACPRs is available, not only to allow practice to be audited to inform education in this area, but also to ensure that accurate data can be used in response to media and anecdotal reporting of DNACPRs which is often misleading and can frighten patients who may benefit from such decisions. We also agree with the CQC report that highlighted the importance of having vigorous oversight of DNACPRs which can be monitored at a local level.^74^ It is crucial that such audits occur, that DNACPR decisions are reviewed regularly at an individual level, and that patients and clinicians are clear about when they can, and should, be used as part of advanced planning and best interests assessments, and how DNACPR decisions can be challenged. This is particularly important given that most people do not understand how a DNACPR works, yet many are seeking protection from CPR.^75^ As David Oliver has recently argued, we need to be having more discussions about DNACPRs as part of end-of-life planning, not fewer.^76^ We therefore welcome the news that a ministerial group has been set up to explore the use of DNACPRs.^77^

## VI. CONCLUSION

This article has discussed the clinical and legal basis for DNACPR decisions and assessed clinical practice across 23 English NHS Trusts before and during the Covid-19 pandemic. Our analysis was based on some 126,545 patients with a DNACPR. Overall, we found that the number of patients with DNACPRs increased during the two Covid-19 waves in 2020–2021, and that these increases were most noticeable in the first wave and affected middle-aged groups more than the very old or very young, though the proportion of patients with a DNACPR in each age group remained broadly consistent across the four data periods. Contrary to widespread public and media concerns about unlawful blanket DNACPR decisions based on age or intellectual disability, we did not find disproportionate increases in DNACPRs for older adults or those with diagnosed learning disabilities during the pandemic. We found, however, that Black Caribbean patients were over-represented in the sample of DNACPRs we analysed, which requires further examination. We also found that, on average, a fifth of patients were not consulted about the DNACPR decision on their medical record. This raises serious concerns about whether there have been human rights breaches and inadequate best interests assessments. However, it is important to note that during the two Covid waves it appears that *more* patients were consulted than in the two pre-Covid periods we analysed. Although we do not have information on whether families were consulted before and during Covid, this finding is stark when compared with other sector surveys and academic research and requires urgent examination. The furore over DNACPRs during Covid has highlighted their controversial nature. It is crucial, looking ahead, that the public has confidence in the use of DNACPRs and that the human rights of patients and their families are protected. This requires full consultation with patients and their families, and redoubled efforts to ensure that DNACPR decisions are not used in a discriminatory way. 

## Footnotes

1 The terms ‘do not resuscitate’ (DNR) and ‘do not attempt Cardio-pulmonary Resuscitation’ (DNACPR) are both used, interchangeably, in academic literature and policy, alongside the terms ‘order’, ‘notice’, and ‘decision’. In this article, we adopt the term DNACPR decision to reflect the fact that a decision has been made by someone—either the patient, a doctor, or medical team (or combination of these) to *not* perform CPR at a future date if a clinical situation occurs where CPR is warranted.

2 NHS, ‘Do not Attempt Cardiopulmonary Resuscitation (DNACPR) Decisions’ (NHS 2021) <https://www.nhs.uk/conditions/do-not-attempt-cardiopulmonary-resuscitation-dnacpr-decisions/> accessed 17 May 2021.

3 NHS (n 2). Although a recent evaluation of mechanical and manual CPR in 58 Covid-19 patients at a single US hospital reports that CPR was successful in 60% of patients, only 22.4% were discharged alive from hospital: A Bhardwaj and others, ‘Effectiveness of Mechanical Cardiopulmonary Resuscitation for Patients with COVID-19 and In Hospital Cardiac Arrest’ (2021) 162 Resuscitation 268.

4 NHS (n 2).

5 JJ Coleman and others, ‘Bringing into Focus Treatment Limitation and DNACPR Decisions: How COVID-19 Has Changed Practice’ (2020) 155 Resuscitation 172, 173.

6 ibid 172.

7 NHS (n 2).

8 D Oliver, ‘David Oliver: Improving DNACPR Discussions, Decisions, and Documentation’ (2021) BMJ 372 [n772].

9 ibid 372.

10
*R (Tracey* *) v Cambridge University Hospitals* [2014] EWCA Civ 33 [2].

11 BBC, ‘Covid-19: Concern over “Do not Resuscitate” Decisions during Pandemic’ (*BBC*, 18 March 2021) <https://www.bbc.co.uk/news/health-56435428> accessed 17 May 2021.

12 J Tapper, ‘Fury at “Do not Resuscitate” Notices given to Covid Patients with Learning Disabilities’ *The Guardian* (London, 13 February 2021) <https://www.theguardian.com/world/2021/feb/13/new-do-not-resuscitate-orders-imposed-on-covid-19-patients-with-learning-difficulties> accessed 17 May 2021.

13 Compassion in Dying, ‘Better Understanding, Better Outcomes: What We’ve Learned about DNACPR Decisions before and during the Coronavirus Pandemic’ (Compassion in Dying, 2021) <https://compassionindying.org.uk/wp-content/uploads/2021/03/Better-Understanding-Better-Outcomes-DNACPR-decisions-before-and-during-the-pandemic.pdf> accessed 17 May 2021.

14 ibid.

15
*R (Tracey) v Cambridge University Hospitals* [2014] EWCA Civ 33.

16 J Samanta, ‘Tracey and Respect for Autonomy: Will the Promise Be Delivered?’ (2015) 23 Medical Law Review 467.

17 ibid [53].

18 ibid [54].

19
*Winspear v City Hospitals Sunderland NHS Foundation Trust* [2015] EWHC 3250 (QB).

20 ibid [45].

21 M Macfarlane and others, ‘How the *Tracey* Judgement Has Changed DNACPR Discussions in Palliative Care’ (2018) 8 BMJ Support and Palliative Care A43.

22 Z Fritz, AM Slowther and G Perkins, ‘Resuscitation Policy Should Focus on the Patient, not the Decision’ (2017) 356 BMJ 1.

23 NHS (n 2).

24 Tapper (n 12).

25 NHS (n 2).

26 A Samuels, ‘Do not Resuscitate: Lawful or Unlawful?’ (2016) 84 Medico-Legal Journal 191.

27 K Liddell and others, ‘Who Gets the Ventilator? Important Legal Rights in a Pandemic’ (2020) 46 Journal of Medical Ethics 421.

28 Care Quality Commission (CQC), ‘Protect, Respect, Connect—Decisions about Living and Dying well during Covid-19’ (London, CWC, CQC 2021).

29 ibid 17.

30 CQC (n 28) 41.

31 For example, R Harwood, ‘Did the UK Response to the COVID-19 Pandemic Fail Frail Older People?’ (*British Geriatrics Society*, 14 May 2020) <https://www.bgs.org.uk/blog/didthe-uk-response-to-thecovid-19-pandemic-fail-frailolder-people> accessed 5 May 2021.

32 Queen's Nursing Institute, ‘The Experience of Care Home Staff during Covid-19 A Survey Report by the QNI's International Community Nursing Observatory’ (Queen's Nursing Institute 2020).

33 ibid 5.

34 British Institute of Human Rights, *Scared, Angry, Discriminatory, Out of**M**y Control: DNAR Decision-Making in 2020* (British Institute of Human Rights 2020).

35 ibid 9.

36 Compassion in Dying (n 13) 4.

37 S O’Keeffe, ‘COVID-19 Pandemic and Decision-Making about Cardiopulmonary Resuscitation and Advance Care Planning’ (2020) 26 Medico-Legal Journal of Ireland 57.

38 Compassion in Dying (n 13).

39 For example, GB Egelund and others, ‘Do-not-Resuscitate Orders in Patients with Community-Acquired Pneumonia: A Retrospective Study’ (2020) 20 BMC Pulmonary Medicine 201; NN Sarkari and others, ‘Impact of Early Do-not-Attempt-Resuscitation Orders on Procedures and Outcomes of Severe Sepsis’ (2016) 36 Journal of Critical Care 134.

40 L Sutton and others, ‘Do Not Attempt Resuscitation (DNAR) Status in People with Suspected COVID-19: Secondary Analysis of the PRIEST Observational Cohort Study’ (2021) 164 Resuscitation 130.

41 E Shepherd and E Ennion, ‘How Has the Implementation of the UK Freedom of Information Act 2000 Affected Archives and Records Management Services?’ (2007) 17 Records Management 32, 33.

42 AJ Fowler and others, ‘The UK Freedom of Information Act (2000) in Healthcare Research: A Systematic Review’ (2013) 3 BMJ Open 2.

43 H Bows, ‘Researching Sexual Violence against Older People: Reflecting on the Use of Freedom of Information Requests in a Feminist Study’ (2017) 115 Feminist Review 30.

44 The government recommends 18 ethnic groups are used to capture an individual’s ethnicity for official purposes. These are also the groups used in the Census of England and Wales. See <https://www.ethnicity-facts-figures.service.gov.uk/style-guide/ethnic-groups> accessed 1 November 2021

45 K Walby and A Luscombe, ‘Ethics Review and Freedom of Information Requests in Qualitative Research’ (2018) 14 Research Ethics 1.

46 L Harrington, K Price and P Edmonds, ‘From Paper to Paperless: Do Electronic Systems Ensure Safe and Effective Communication and Documentation of DNACPR Decisions?’ (2020) 20 Clinical Medicine 329, 329.

47 NHS England, ‘Five Year Forward View’ (NHS 2014) <https://www.england.nhs.uk/wp-content/uploads/2014/10/5yfv-web.pdf> accessed 18 May 2021.

48 Data provided through FOI requests become public data and are often (but not always) published by the data owner (in this case, each NHS Trust) in an online log. The anonymised data from our study has been uploaded to the Durham Research Online DATAsets Archive doi:10.15128/r1mw22v549s.

49 Coleman and others (n 5).

50 Office for National Statistics (ONS), ‘UK Population Pyramid Interactive’ <https://www.ons.gov.uk/peoplepopulationandcommunity/populationandmigration/populationestimates/articles/ukpopulationpyramidinteractive/2020-01-08> accessed 17 May 2021.

51 Office for National Statistics (ONS), ‘Estimates of the Very Old, Including Centenarians, UK: 2002 to 2019’ (ONS 2020) <https://www.ons.gov.uk/peoplepopulationandcommunity/birthsdeathsandmarriages/ageing/bulletins/estimatesoftheveryoldincludingcentenarians/2002to2019> accessed 18 May 2021.

52 Coleman and others (n 5).

53 ibid.

54 British Medical Association, Resuscitation Council (UK) and Royal College of Nursing, *Decisions Relating to**Cardiopulmonary**Resuscitation Council* (3rd edn, 2016) <https://www.resus.org.uk/sites/default/files/2020-05/20160123%20Decisions%20Relating%20to%20CPR%20-%202016.pdf> accessed 25 August 2021.

55 UK Government, ‘Population of England and Wales’ (Gov.uk, 7 August 2020) <https://www.ethnicity-facts-figures.service.gov.uk/uk-population-by-ethnicity/national-and-regional-populations/population-of-england-and-wales/> accessed 29 October 2021.

56 CQC (n 28).

57 L Sutton and others, ‘Do not Attempt Cardiopulmonary Resuscitation (DNACPR) Decisions in People Admitted with Suspected COVID-19: Secondary Analysis of the PRIEST Observational Cohort Study’ (2021) 164 *Resuscitation* 130.

58 Department of Health and Social Care (DHSC), The NHS Constitution (DHSC, 2021).

59 CQC (n 28) 23–24.

60 ibid 18.

61 General Medical Council, ‘Treatment and Care towards the End of Life: Good Practice in Decision Making’ (London, 20 May 2010) <https://www.gmc-uk.org/-/media/documents/treatment-and-care-towards-the-end-of-life–-english-1015_pdf-48902105.pdf> accessed 12 November 2021.

62 It should be noted that this was based on the perceptions and information held by social care providers who responded to a request for information. Thus, the figure of 5.2% (508 cases) could not be independently verified.

63 Coleman and others (n 5).

64 M Tinkler and K Millington, ‘Do not Attempt Resuscitation Decisions (DNACPR): Do We Fulfil the Human Rights Act?’ (2016) 48 European Respiratory Journal PA3741.

65
*R (Tracey) v Cambridge University Hospitals* [2014] EWCA Civ 33.

66
*Duce v Worcestershire Acute Hospitals NHS Trust [*2018] EWCA Civ 1307.

67
*County Durham & Darlington NHS Foundation Trust v PP* [2014] EWCOP 4 [51].

68 NHS (n 2).

69
*NHS Cumbria CCG v Rushton* [2018] EWCOP 41.

70
*Aintree University Hospital NHS Foundation Trust v James* [2013] UKSC 67, [39].

71 NHS, *NHS Long Term Plan* (NHS 2019).

72 ibid.

73 Harrington and others (n 46) 333.

74 CQC (n 28) 7.

75 Compassion in Dying (n 13).

76 Oliver (n 8).

77 They Work For You, ‘DNACPR Decisions: Coronavirus: Department of Health and Social Care Written Question – Answered on 14th June 2021’ (14 June 2021) <https://www.theyworkforyou.com/wrans/?id=2021-06-04.10666.h> accessed 6 September 2021.

